# Recent advances in fungal xenobiotic metabolism: enzymes and applications

**DOI:** 10.1007/s11274-023-03737-7

**Published:** 2023-09-02

**Authors:** Mohd Faheem Khan, Carina Hof, Patricie Niemcová, Cormac D. Murphy

**Affiliations:** UCD School of Biomolecular and Biomedical Science, University College Dublin, Belfield, Dublin 4, Ireland

**Keywords:** Biodegradation, Biotransformation, Lignin-degrading enzyme, Organic compounds, Pollutant

## Abstract

Fungi have been extensively studied for their capacity to biotransform a wide range of natural and xenobiotic compounds. This versatility is a reflection of the broad substrate specificity of fungal enzymes such as laccases, peroxidases and cytochromes P450, which are involved in these reactions. This review gives an account of recent advances in the understanding of fungal metabolism of drugs and pollutants such as dyes, agrochemicals and per- and poly-fluorinated alkyl substances (PFAS), and describes the key enzymes involved in xenobiotic biotransformation. The potential of fungi and their enzymes in the bioremediation of polluted environments and in the biocatalytic production of important compounds is also discussed.

## Introduction

Xenobiotics are compounds that are not naturally produced or normally found in living systems and include agrochemicals, drugs, cosmetics and industrial chemicals, and prolonged exposure to them can have severe health consequences. Our reliance on xenobiotic compounds to drive our economies, ensure sufficient food production and improve our health results in increasing pollution concerns, as many of these compounds are not naturally rapidly degraded in the environment nor are they removed in wastewater treatment facilities (Štefanac et al. 2021). Various physicochemical methods have been developed for the remediation of xenobiotic pollution, including adsorption, precipitation, chemical oxidation and membrane separation; however, biological approaches employing plants and microorganisms, are viewed as being more sustainable (Sharma et al. 2023), thus much research is devoted to sustainably produce and degrade xenobiotics. Fungi play a key role in the decomposition of dead organic matter, thus are widely investigated for their bioremediation potential, since they have a suite of enzymes than enable the biotransformation of a very broad range of compounds (Chen et al. 2022; Zhuo and Fan 2021).

The production of important industrial compounds involves hazardous reagents and often high temperatures and pressures, leaving a large environmental footprint (Kim and Li 2020). In contrast, enzymatic reactions are conducted under mild conditions of pH and temperature and do not typically involve dangerous reagents, thus biocatalysis is attractive as an alternative to classical chemical synthesis. Fungal enzymes that catalyse oxidative reactions are highly useful in the oxyfunctionalisation of organic substrates, thus have potential in the sustainable production of commercially valuable compounds (Aranda et al. 2021).

In this review, the important discoveries in the field of fungal xenobiotic metabolism over the last decade are summarised, along with the key enzymes involved and the applications for bioremediation and biocatalysis. The paper deals with recent examples of the different types of xenobiotics that are catabolised by fungi (drugs, pesticides, dyes, dyes, polyaromatic hydrocarbons, halogenated pollutants and plastics) and the key enzymes that are responsible for the biotransformations (CYPs, peroxidases, laccases, tyrosinases and unspecific peroxygenases). In the Outlook section, the potential and limitations of fungi in bioremediation and biocatalysis are discussed.

## Classes of xenobiotics catabolised by fungi

### Drugs

**Fig. 1:**
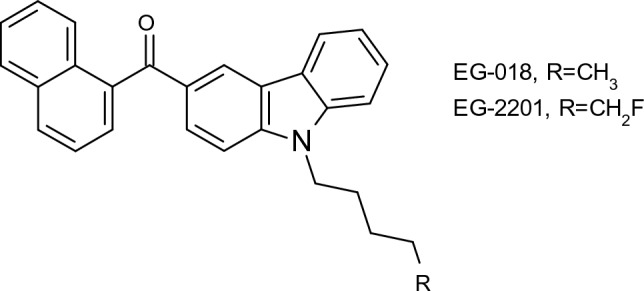
Structures of the synthetic cannabinoids EG-018 and EG-2201

Researchers have for decades studied the biotransformation of drugs using fungi. Early studies by Smith and Rosazza (1974) demonstrated that fungi, including *Penicillium chrysogenum*, *Aspergillus niger* and *Cunninghamella bainieri*, hydroxylated a series of model aromatic compounds including acetanilide, acronycine, coumarin and naphthalene. In the study it was also proposed that fungi might be used as models of mammalian xenobiotic metabolism and could be applied to the production of important metabolites at scale. Research into this aspect of fungal xenobiotic catabolism has continued since then and has been reviewed regularly (Asha and Vidyavathi 2009; Goncalves et al. 2021; Murphy 2015). Typically, researchers focus on fungi belonging to the genera *Cunninghamella*, *Mucor*, *Aspergillus* and *Fusarium*, as these fungi have enzymes, such as cytochromes P450 (CYPs), sulfotransferases and glycosyltransferases, that catalyse phase I (oxidative) and phase II (conjugative) reactions that are part of drug detoxification in humans. Conventional experiments to investigate fungal biotransformation of drugs involves growth of the fungi on a standard growth medium such as Sabauroud dextrose or potato dextrose, and the cultures are incubated with the drug under investigation. The metabolites are extracted from the culture with a suitable organic solvent and are analysed using gas chromatography- (GC-) and liquid chromatography (LC)-mass spectrometry (MS), and nuclear magnetic resonance (NMR) spectroscopy. Often researchers will also conduct parallel investigations with human liver microsomes to compare the metabolites formed from the fungal biotransformations to those that are formed in humans. In a recent study, Gaunitz et al. (2019) compared the phase I metabolism of the synthetic cannabinoids EG-018 (naphthalen-1-yl(9-pentyl-9 H-carbazol-3-yl)methanone) and its fluorinated analogue EG-2201 (Fig. 1) by human liver microsomes, CYP isozymes and *Cunninghamella elegans*. Microsomal incubation resulted in the formation of 15 phase I metabolites from EG-018 and 21 from EG-2201 arising mainly from hydroxylations on the pentyl arm, and the carbazole and naphthyl rings. The fungal metabolites produced from the two substrates correlated well with those produced from microsomes, illustrating that in addition to fungal enzymes catalysing analogous reactions to mammalian enzymes, they can also biotransform fluorinated substrates. Many anthropogenic compounds are fluorinated and the importance of fungi in their biotransformation will be discussed further below.

More recently fungal unspecific peroxygenases (UPOs), which are related to CYPs but use H_2_O_2_ as the oxidant, have been shown to efficiently biotransform drugs into mammalian metabolites (Kinner et al. 2021). Owing to the introduction of the Metabolites in Safety Testing (MIST) regulations by the Food and Drug Administration (FDA), access to drug metabolites has become increasingly important so that their toxicity profile can be determined (Schadt et al. 2018). Microorganisms such as fungi are readily scalable, thus they are potentially a sustainable alternative to chemical synthesis for producing drug metabolites (Klenk et al. 2019).

**Fig. 2:**
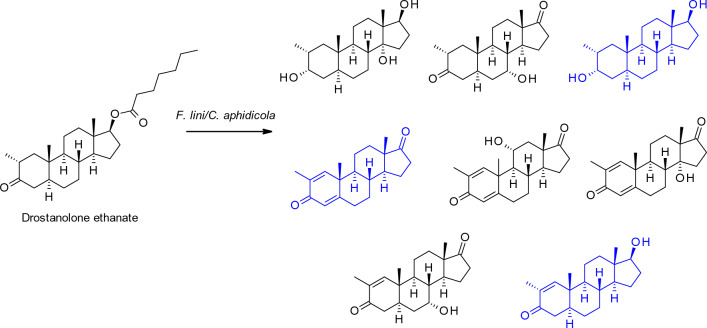
Biotransformation of drostanolone ethanate by *F. lini* and *C. aphidicola* generates nine metabolites: The compounds in blue were previously known and the remainder were newly discovered (Choudhary et al. 2017)

A second major reason for investigating fungal biotransformation of drugs is to produce metabolites that are different to those observed in mammals, and which have modified bioactivity and are themselves potential drug leads. For example, Choudhary et al. (2017) employed the fungi *Cephalosporium aphidicola* and *Fusarium lini* to biotransform the anticancer steroid drug drostanolone enanthate, yielding eight products through de-esterification, carbonyl reduction, dehydrogenation and hydroxylation (Fig. 2). Five of the metabolites were previously unknown and all of the metabolites were assessed for anticancer activity against a range of cell lines; several of the metabolites were more active against HeLa and PC-3 cell lines than the original drug.

Some researchers have explored how fungal biofilms might be applied to the improved production of drug metabolites. Biofilms are a natural form of immobilisation and provide cells with mechanical stability and increased resistance to toxic compounds (Harding et al. 2009). Amadio et al. (2013) and Quinn et al. (2015) reported that biofilms of *C. elegans* that were cultivated easily in Erlenmeyer flasks containing a steel spring can be easily re-used for the semi-continuous production of phase I metabolites of drugs such as flurbiprofen and diclofenac. Bianchini et al. (2021) compared the biotransformation of diclofenac to the main mammalian metabolite 4’-hydroxydiclofenac by *C. elegans* in a conventional air-lift reactor (ALR), bubble column reactor (BCR) and a hybrid fixed bed air-lift reactor (FB-ALR). The latter vessel promoted the formation of biofilm, and this facilitated an improved metabolite production (approx. 30% yield) compared with other reactors, which yielded approx. 18% (ALR) and 12% (BCR).

## Pesticides

Microorganisms have been studied extensively for their ability to degrade pesticides (Ayilara and Babalola 2023) and the same fungi described above are often employed in pesticide biotransformation and biodegradation studies. For example, *C. elegans* has been employed to degrade a range of pesticides including the pyrethroids cyhalothrin, transfluthrin and β-cyfluthrin (Khan and Murphy 2021; Palmer-Brown et al. 2019), and the organophosphate pesticides fenitrothrion and diazinon (Zhao et al. 2020; Zhu et al. 2017). The degree of catabolism varies depending on the nature of the pesticide and the fungus employed, thus it is possible that in some cases metabolites that are potentially toxic can be produced. For example, in the case of λ-cyhalothrin, which contains a trifluoromethyl group, there were numerous metabolites detected upon incubation with *C. elegans*, but no fluoride ion in the aqueous fractions could be measured, indicating that the fluorine-containing portion of the molecule was not fully catabolised (Palmer-Brown et al. 2019). *Aspergillus sydowii* and *Penicillium decaturense* degraded the organophosphate pesticide methyl parathion when cultured in malt broth for 20 and 30 days, respectively (Alvarenga et al. 2014). The substrate was initially converted to methyl paraoxon then to *p*-nitrophenol. Bhatt et al. (2020) isolated a strain of *F. proliferatum* that could grow on high concentrations (1000 mg/L) of the pesticide allethrin as a sole carbon and energy source. However, the biodegradation pathway was not investigated. 3,4-Dinitroaniline is a prominent pollutant arising from phenylurea herbicides can be successfully detoxified by fungi such as *Aspergillus niveus*, *Aspergillus terreus*, and *Cladosporium cladosporioides*. Rodrigues et al. (2023) demonstrated that these fungi catalysed the *N*-acetylation of the substrate, resulting in reduced toxicity towards plants seeds and human cells. A summary of some recent examples is shown in Table 1.

**Table 1:** Recent examples of pesticide biotransformation by fungi

Pesticide	Class	Structure	Fungus	Key enzymes	Reference
Diuron	Aryl urea		*T. versicolor*	CYP	Hu et al. (2020)
Alachlor	Chloroacetanilide		*Trichoderma* spp.	N.D.*	Nykiel-Szymanska et al. (2020)
Nitenpyram	Neonicotinoid		*Phanerochaete sordida*	CYP	Wang et al. (2019b)
Cyhalothrin	Pyrethroid		*C. elegans*	CYP	Palmer-Brown et al. (2019)
Fenitrothion	Organophosphate		*C. elegans*	N.D.	Zhu et al. (2017)
Propazine	Chlorotriazine		*P. ostreatus*	Laccase, CYP	Pereira et al. (2016)
Trans-chlordane	Organochlorine		*Phlebia* spp.	CYP	Xiao et al. (2011)

*N.*D*. not determined

### Dyes

The textile industry, which is a particularly important commercial activity in developing countries, employs synthetic dyes, which eventually pollute local water courses. Lignin degrading fungi have been intensely investigated for the degradation of azo dyes. Their oxidative enzymes, especially laccase, are the key biocatalysts and generate reactive oxygen species that non-specifically oxidise dyes. In a recent study *F. oxysporium*, a fungus with numerous putative genes coding for laccase, was shown to degrade high concentrations of the dyes aniline blue, reactive black 5, orange II and crystal violet (Thoa et al. 2023). The authors also demonstrated the importance of including 1% glucose, a laccase mediator such as 1-hydroxybenzotriazole and Remazol brilliant blue R (RBBR), which is a laccase inducer, to the degradation of the dyes. Yu et al. (2023) isolated a strain of *A. tabacinus* from a soil sample that could employ the dye Acid Red 73 (AR73) as the sole carbon and nitrogen source for anaerobic growth, employing an unusual ‘self-redox’ mechanism in which the carbon of the substrate was oxidised to CO_2_ and the nitrogen reduced to ammonia. Furthermore, the authors identified an oxygen-sensitive hydrolase (Ord 95) that degraded AR73 and detected the compounds 2-hydroxynaphthalene and *N*-phenylnitrous amide by LC–MS. The same compounds were detected in experiments using whole cells of *A. tabacinus*, thus it was concluded that the enzyme catalysed the C–N= bonds of the dye. The protein had some homology to the N-terminal domain of glutathione *S*-transferase and contains three key arginine residues; it was proposed that the C–N cleavage was coupled with the reduction of oxidised glutathione. Non-lignin-degrading fungi also degrade dyes, for example, *C. elegans* can degrade the triphenylmethane dye malachite green to leucomalachite green and other metabolites, via its CYP activity (Cha et al. 2001). Hexavalent chromium is commonly found in regions contaminated with textile dyes, so Hussain et al. (2017) investigated if this fungus could simultaneously remove the dye and metal from water and found that *C. elegans* biofilms could remove these contaminants in a semi-continuous manner. The biofilm could remove 95% of dye and metal for 19 repeated additions. Other fungal biofilms have also shown improved dye removal compared with non-immobilised cells. Biofilm of *Coriolopsis* sp. 1c3 grown on a muslin cloth degraded cotton blue and crystal violet more effectively than suspended cells (Munck et al. 2018). Similarly, *A. flavus* A5p1 immobilised on polyurethane foam was more effective at degrading reactive blue 4 compared with suspended cells, and in a packed-bed reactor over 90% decolourisation was maintained over 26 days (Yang et al. 2022).

### Polyaromatic hydrocarbons

Polyaromatic hydrocarbons (PAHs) are non-polar organic compounds that have multiple rings of various sizes, which arise from burning of fossil fuels and wood. They are carcinogenic and consequently much research has been done on the bioremediation of PAH-contaminated environments using microorganisms. Fungi have been extensively investigated for their ability to degrade PAHs, mainly those with fewer than six rings, and the topic has been recently reviewed elsewhere (Elyamine et al. 2021; Gupta and Pathak 2020; Kadri et al. 2017) so only a brief overview will be provided here. Typical biodegradation pathways employ either lignin-degrading enzymes (Mn peroxidase and lignin peroxidase) to generate quinones from PAHs enabling ring-opening and eventual catabolism to CO_2_, or CYPs that initially oxidise the substrate yielding an epoxide that can either spontaneously form a phenol or be enzymatically converted into a dihydrodiol. Park et al. (2019) analysed the transcriptome of the white rot fungus *Dentipellis* sp. KUC8613 upon incubation with the PAHs anthracene, fluoranthene, phenanthrene and pyrene. Unexpectedly the lignolytic genes were not upregulated in these experiments, but 15 different CYP genes were upregulated depending on the PAH present. Twenty-seven other genes were upregulated by the PAHs, including hydrolases, alcohol dehydrogenases, aldehyde dehydrogenases, monooxygenases, dioxygenases, glutathione-*S*-transferases and a sulfotransferase, which were likely to be involved in the downstream catabolism of the substrates.

Degradation of PAH in soil has been shown to be improved by bioaugmentation with fungi. For example, Baldantoni et al. (2017) conducted mesocosm experiments in which the degradation of benzo[a]pyrene and anthracene was measured in soil that was supplemented with either compost or a fungal consortium (*Armillaria mellea, Pleurotus ostreatus, Pleurotus eryngii*, and *Stropharia ferii*). Compared with untreated soil, PAH degradation was faster in the treated soils, and the fungal consortium was best at degrading anthracene. Soil pH is another factor which influences the fungal degradation of PAH: Vipotnik et al. (2021) reported that in experiments with *Trichoderma viride*, *Penicillium chrysogenum* and *Agrocybe aegerita* a soil pH of 5 resulted in a better degradation of fluorene, pyrene and benzo[a]pyrene than pH 7, whereas biodegradation of chrysene was better at pH 7. Low bioavailability of PAHs in soils can also limit their biodegradation. To address this issue, Wang et al. (2021) applied rhamnolipid from *Pseudomonas aeruginosa* as a biosurfactant and agricultural waste to PAH contaminated soil. The combination of rhamnolipid and agricultural waste significantly improved PAH removal by up to 20% by increasing the dissolved organic carbon and improving the PAH bioavailability. Microbial community analysis demonstrated an increased abundance of PAH-degrading fungi from the genera *Humicola, Gibberella, Chaetomium, Thielavia*, and *Mortierella* in addition to bacteria known to degrade these compounds.

Mitra et al. (2013) demonstrated that *C. elegans* biofilm grown in a polymethylmethacrylate conico-cylindrical flask (PMMA-CCF) degraded fluoranthrene 22-times more effectively than suspended culture. Confocal laser scanning microscopy showed co-localisation of fluoranthrene within the extracellular polymeric substance enhancing biotransformation. A mixed biofilm of the yeasts *Candida viswanathii* TH1, *Candida tropicalis* TH4 and *Trichosporon asahii* B1, that were isolated from oil-contaminated environments, degraded a mixture of PAHs (naphthalene, anthracene and pyrene, 200 ppm each) and phenol (600 ppm) over 7 days (Cong et al. 2014).

### Halogenated pollutants

Heavily halogenated pollutants such as polychlorinated biphenyls (PCBs) and per- and polyfluorinated alkyl substances (PFAS) are difficult to biodegrade. Nevertheless, there are numerous examples of fungi that can biotransform these compounds, providing a bioremediation route to contaminated environments by generating metabolites that, if not easily degraded by the fungus itself, could be degraded by other microorganisms that might be present. *Pleurotus ostreatus* in particular is a highly effective PCB degrader (Chun et al. 2019) as it degraded over 99% of the PCBs in 1 mg/L Delor 103, which is a mixture of over 25 congeners with an average of three chlorosubstituents (Čvančarová et al. 2012). Numerous metabolites were detected by GC-MS, including chlorinated hydroxy- and methoxy-biphenyls, along with chloro-benzoic acids, -benzaldehydes and -benzyl alcohols. Fungal laccases are known to degrade hydroxylated chlorinated biphenyls in vitro (Kordon et al. 2010; Sredlova et al. 2021), but their precise role in PCB degradation in vivo is unclear. For instance, some studies have shown an increase in laccase activity and expression in the presence of PCBs (Gayosso-Canales et al. 2012; Sadanoski et al. 2019, 2020), whereas others have observed no correlation between laccase expression and PCB degradation (Perigon et al. 2019). Plackova et al. (2012) reported that in non-growing *T. versicolor* there was no PCB degradation despite a 2.6-fold increase in laccase expression. Germain et al. (2021) isolated 12 fungal strains from PCB-contaminated soil and studied six effective PCB degraders (three *Penicillium* spp., *Aspergillus jensenii, Acremonium sclerotigenum* and *Trametes versicolor*). The fungi could degrade PCB congeners containing three to six chlorine atoms, and laccase activity in *T. versicolor* and peroxidase activity in *A. sclerotigenum* was induced by PCBs. However, by assessing the toxicity of the metabolites produced by the different fungi, it was concluded that the strains had different degradation pathways, so the precise role of the different enzymes in PCB degradation is unclear.

PFAS are environmental contaminants of immediate concern, as they are used in numerous everyday products, such as food packaging, non-stick cookware and stain-resistant fabrics, and in a range of other applications such as fire-fighting foams and electroplating (Glüge et al. 2020). PFAS are hazardous to human health and diseases including cancer, pregnancy-induced high blood pressure, ulcerative colitis and high cholesterol being linked to exposure (Sunderland et al. 2019). There are limited studies investigating the biotransformation and biodegradation of PFAS in fungi, nevertheless, some important findings have been reported. Several fungi have been identified that biotransform 6:2 fluorotelomer alcohol (6:2 FTOH, C_6_F_13_CH_2_CH_2_OH). Tseng et al. (2014) discovered that *P. chrysogenum* degraded this compound over 28 days to a number of per- and polyfluorinated carboxylic acids, with 5:3 fluorotelomer carboxylic acid (5:3 FTCA, C_5_F_11_CH_2_CH_2_COOH) being the most abundant. Merino et al. (2018) isolated additional fungal strains from soil contaminated with PFAS -containing fire-fighting foam and investigated them for 6:2 FTOH biotransformation alongside two other fungi *Gloeophyllum trabeum* and *T. versicolor*, and detected similar products. Khan and Murphy (2023b) observed that *C. elegans* efficiently degraded higher concentrations (100 mg/L) of 6:2 FTOH in 48 h, also detecting 5:3 FTCA as the main metabolite. Additionally, it was shown that this metabolite is inhibitory and that CYP activity is crucial to the degradation of 6:2 FTOH in this fungus. Most recently Merino et al. (2023) demonstrated the involvement of CYPs in the biotransformation of 6:2 FTOH in *P. chrysogenum* by measuring overall cytochrome P450 reductase activity (CPR) and monitoring CYP gene expression in the presence of 6:2 FTOH and its metabolites. However, the exact enzymes involved in fungal 6:2 FTOH degradation are not known, and only one report of an in vitro biotransformation of 6:2 FTOH to 6:2 FTCA, by a heterologously expressed CYP from *C. elegans*, has been reported (Khan and Murphy 2022). While these discoveries are of high interest, the contribution of fungi to the degradation of PFAS in the soil is not fully known, but they nevertheless indicate the potential of some fungi that might be applied to specific areas of PFAS contamination, such as areas where PFAS-containing firefighting foams are used heavily (Dong et al. 2023).

Pentachlorophenol, which is used as a biocide and wood preservative, is degraded by the fungus *Phlebia acanthocystis*, yielding methylated and oxidised products such as pentachlorophenol anisole and *p*-tetrachlorohydroquinone (Xiao and Kondo 2020). Extracellular peroxidases (lignin peroxidase and Mn peroxidase) were demonstrated to be involved in pentachlorophenol biotransformation via in vitro experiments with extracellular fluid, and inhibitor experiments showed the importance of intracellular CYPs in the oxidation of the substrate to *p*-tetrachlorohydroquinone.

### Plastics

**Fig. 3:**
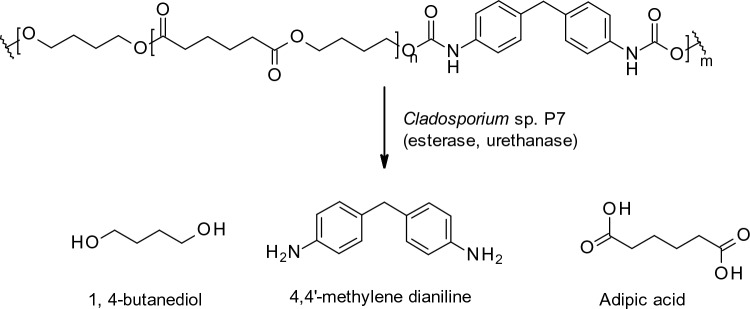
Degradation of PBA-PU by *Cladosporium* sp. P7. The initial products of esterase and urethanase activity were further degraded by the fungus (Liu et al. 2023)

Global production of plastic was over 309 million metric tons in 2021 (www.statista.com/statistics/282732/global-production-of-plastics-since-1950/), the bulk of which is eventually buried in landfill, with consequential ecotoxicological effects caused by the plasticisers, additives and co-polymers (Geyer et al. 2017). The ecological damage caused by plastics such as polyethylene (PE), polyurethane (PU) and polyethylene terephthalate (PET) has prompted much research on the biodegradation of these polymers by microorganisms. Species of fungi belonging to *Aspergillus*, *Pleurotus*, *Penicillium* and *Cladosporium* have been identified as plastic degraders and their potential for plastic bioremediation has been reviewed very recently by several groups (Bhavsar et al. 2023; Solanki et al. 2022; Srikanth et al. 2022). In addition to expressing key oxidoreductases and hydrolases, which shorten the polymeric chain, fungi produce surface active proteins called hydrophobins that not only enable adhesion to hydrophobic surfaces but also directly interact with the depolymerisation enzymes (Zhang et al. 2022). Most recently, Liu et al. (2023) isolated 20 microbial strains capable of degrading poly(1,4-butylene adipate)-based PU (PBA-PU) as the screening substrate. Agar plates containing this compound were opaque, so isolates that could degrade the compound were identified via zones of clearing. The fungus *Cladosporium* sp. 7 was the most effective PBA-PU degrader and could degrade two other polyurethane polymers: Impranil DLN-SD and PU foam. HPLC, GC and mass spectral analysis revealed adipic acid, 1, 4-butanediol and 4,4’-methylenedianiline as initial products of PBA-PU degradation (Fig. 3), which arise from the hydrolysis of the ester and urethane bonds. These metabolites were further degraded in the fungus as carbon and energy sources. Taxeidis et al. (2023) developed an extended screening method to identify PU- and PE-degrading fungi by evaluating strains that were isolated from a range of environmental sources firstly on minimal medium agar plates containing Impranil DLN-SD as a carbon source to identify potential PU-degraders. The strains were assessed for their potential as PE degraders by inoculating on minimal medium agar plates containing a mixture of long chain alkanes (tetrasocane, octasocane and hexatriacontane). The extracellular enzymes from three isolates, one *Aspergillus* and two *Fusarium* species, were investigated further for their ability to degrade PU and low-density polyethylene (LDPE). Esterase activity correlated with the degradation of PU in *Fusarium*, strongly suggesting that these enzymes are involved in PU degradation, whereas in the LDPE-degrading *Aspergillus* isolate, proteomic analysis indicated a role for FAD-dependent oxidoreductase. Di Napoli et al. (2023) detected oxidoreductase activity in *C. haloterans* which was isolated from the gastric system of *Galleria mellonella* larvae. This fungus degraded high density polyethylene (HDPE) and extracellular oxidoreductase activity was induced by the presence of the plastic. Comparatively high esterase activity was detected in supernatant extracts of *Embarria clematidis* which degraded Impranil to a greater degree (89%) compared with *A. niger* (49%) over a 2 week period.

Fungal enzymes (cutinases, lipases and esterases) also have potential in the recycling of PET by depolymerising the polymer to its monomers ethylene glycol and terephthalic acid (Ahmaditabatabaei et al. 2021), which is important in a circular bioeconomy context. Malafatti-Picca et al. (2023) screened 100 fungi from a collection of strains that were of interest to the petrochemical industry and showed that four of these produced at least 12 ppm terephthalic acid upon incubation with PET nanoparticles and future optimisation of these strains could improve monomer recovery enabling scalable application in PET recycling.

#### Enzymes involved in xenobiotic catabolism

The key to fungi’s ability to degrade a wide variety of xenobiotics is their enzymes. The main enzyme classes involved in xenobiotic biotransformation have been identified in the preceding sections and include CYPs, peroxidases, laccases, tyrosinases, and unspecific peroxygenases. What follows is a description of the state-of-the-art of these essential enzymes.

#### CYPs (EC 1.14.x.x)

Cytochrome P450 (CYP) enzymes are membrane-bound haem-containing monooxygenases that add one oxygen atom from molecular dioxygen (O_2_) to the substrate. In fungi, CYPs play roles in cellular metabolism, adaptation, pathogenicity, decomposition, and as described above, biotransformation of hazardous chemicals. Fungal CYPs are widely distributed among different phyla, where they are involved in a range of cellular processes including secondary metabolite biosynthesis, ergosterol biosynthesis and, most relevant to this review, xenobiotic biotransformation (Lin et al. 2022). Despite sharing conserved motifs, the sequence similarity between CYPs is low, and cluster into 15 clades (Chen et al. 2014). CYP monooxygenases not only catalyse hydroxylation reactions, but also epoxidation, dehalogenation, decarboxylation, demethylation, denitrification, desulfurization, and desaturation, on a broad range of substrates (Gangola et al. 2019), some examples of which are shown in Table 2. Fungal CYP systems can be classified based on their interaction with one or more redox partners, namely (i) three component system or cytochrome P450 reductase (CPR)-cytochrome b5 (cytb5)-CYP, (ii) two component systems (CPR-CYP system), and (iii) one component system (soluble and fused CPR:CYP). All the CYP systems follow a similar catalytic cycle which begins with the transfer of electrons from NADPH (or NADH) to either directly or indirectly to haem of the CYP monooxygenase (Črešnar and Petrič 2011).

**Table 2:** Biotransformation of xenobiotics by fungal CYPs

CYP type(s)	Xenobiotic biotransformation	Fungal source	Reference
CYP51A, CYP51B, CYP51C	Demethylation of azole fungicides	*A. flavus*	Lucio et al. (2020)
CYP617D1	Hydroxylation of benzo(a)pyrene	*A. nidulans*	Ostrem Loss Erin et al. (2019)
CYP53A15	Hydroxylation of cinnamic acid derivatives	*A. niger*, *P. ostreatus*, *Cochliobolus lunatus*	Korošec et al. (2014)
CYP5208A3, CYP5313D1	Hydroxylation of flurbiprofen, ibuprofen and diclofenac; ester cleavage of transfluthrin and β-cyfluthrin; oxidation of 6:2 FTOH	*C. elegans*	Khan and Murphy (2022)
CYP51A, CYP51B	Demethylation of azole fungicides	*F. graminearum*, *F. oxysporum*	Song et al. (2018); Zheng et al. (2019)
CYP5136A3, CYP63A2	Hydroxylation (ω-oxidation) of endocrine disrupting alkylphenols	*P. chrysosporium*	Syed et al. (2011)
CYP5147A3, CYP5037B3, CYP5147A3	N-dealkylation of acetamiprid, imidacloprid, and thiacloprid	*P. chrysosporium*	Mori et al. (2021); Wang et al. (2019a)
CYP5150D1, CYP5027B1, CYP5350B2v1	Hydroxylation of anthracene, carbazole, phenanthrene, and pyrene	*Postia placenta*	Ide et al. (2012)

**Fig. 4:**
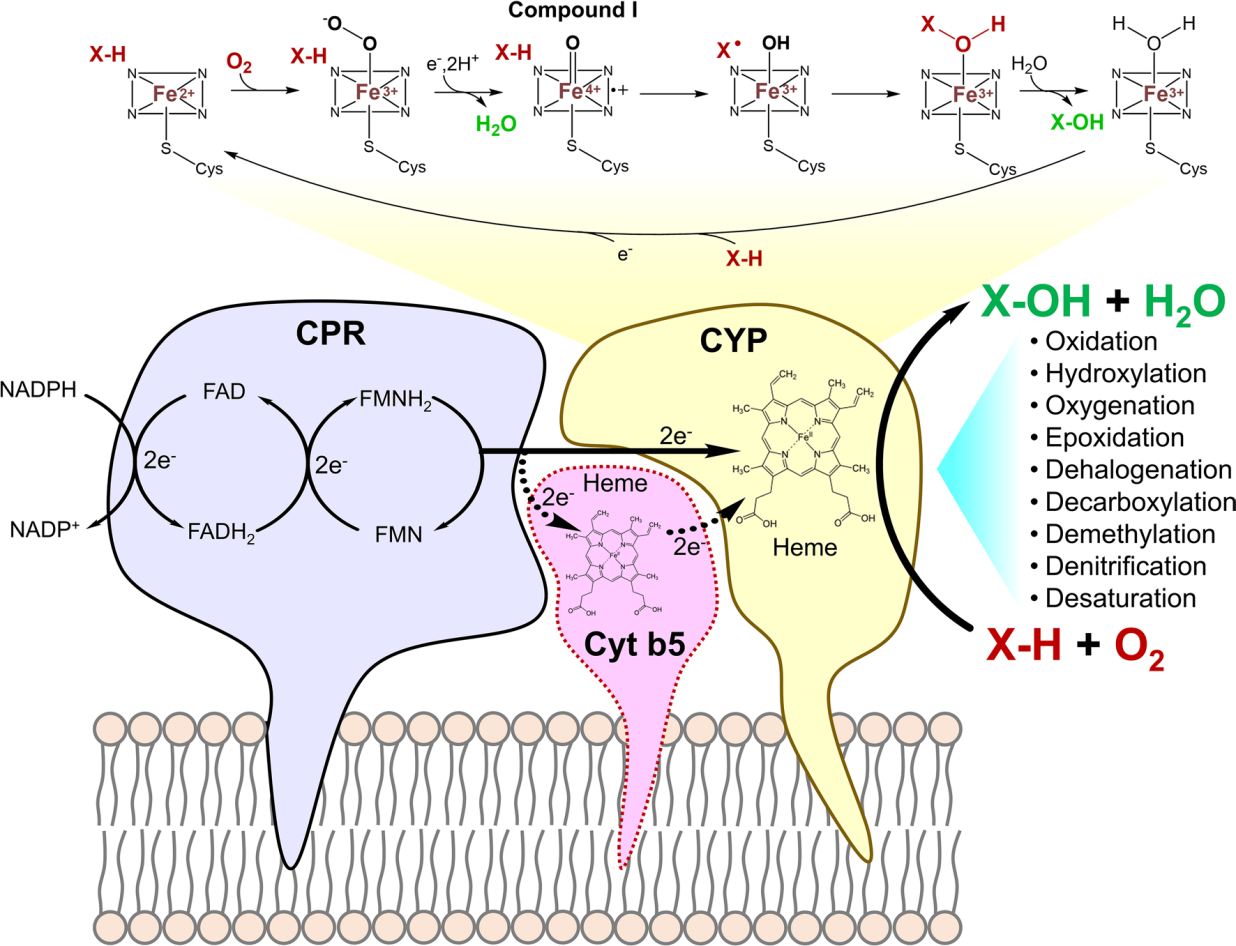
Catalytic mechanism of CYP system involved interaction between CPR, Cyt b5 and CYP monooxygenase in the hydroxylation of substrate X-H

The molecular mechanism of CYP-catalysed monooxygenation has been well described by Durairaj et al. (2016) and Zhang et al. (2021) and is depicted in Fig. 4. In summary, electrons are transferred from NADPH to membrane-bound CPR, activating the membrane-bound CYP by reducing heme-Fe^3+^ to heme-Fe^2+^ through reduction via FAD to FADH (at C-terminal of CPR) and FMN to FMNH_2_ (at N-terminal of CPR). A dioxygen molecule binds to reduced heme-Fe^2+^ to form peroxo-ferric intermediate heme-Fe^3+^–O–O^−^ which upon protonation, generates a reactive ferryl intermediate heme-Fe^4+^=O^+•^ (Compound I) and releases a water molecule. This highly reactive intermediate abstracts a H-atom from the substrate (X-H), producing heme-Fe^4+^–OH and a substrate radical (X^•^), which then rebounds to form heme-Fe^3+^–X-OH complex and yielding the monooxygenated product X-OH, which released upon addition of water.

The increasing availability of fungal genome data, for example through the 1000 fungal genomes project, has enabled researchers to investigate the function of the many different CYPs in important fungi, such as *P. chrysosporium*, through heterologous expression. Various expression systems are employed, but challenges such as production of soluble CYP forms, their localization as membrane proteins, haem group incorporation and coupling with CPRs/cytb5, have been encountered (Jiang et al. 2021). Troubleshooting approaches for producing functional recombinant fungal CYPs involve various strategies such as selecting suitable host organisms, optimizing expression vectors, adjusting cultivation and media conditions, optimizing codons, inducing protein localization into the cytosol through truncation/modification and optimizing coupling with redox partners (Jiang et al. 2021; Nauen et al. 2021; Theron et al. 2014). One recent example of successful heterologous expression of fungal CYPs is that of the screening of the CYPome of *Thamnidium elegans* for valuable biocatalytic activities (Permana et al. 2022). Each of 46 CYP genes in the CYPome was systematically co-expressed with a *T. elegans* CPR in *S. cerevisiae*, and the library screened for activity using steroid and drug substrates. This approach led to the discovery of CYP5312A4, which catalysed a highly unusual 14α-hydroxylation of testosterone.

Khan and Murphy (2022) expressed CYP5208A3 from *C. elegans* in *Pichia pastoris* and demonstrated the importance of the redox partner for CYP activity. *C. elegans* has three CPRs in its genome and these genes were individually co-expressed with the CYP in *P. pastoris*. The monooxygenase activity of the recombinant yeast was assessed by incubation with a range of drugs and pesticides, and it was observed that the optimum biotransformation of the different substrates employed a different combination of CYP and CPR. It was proposed that having more than one CPR enables a multiplicity of CYP-CPR combinations thereby broadening the substrate range that can be biotransformed by the *C. elegans* CYPs and might explain how this fungus is able to catabolise such a wide range of xenobiotics.

### Peroxidases (EC 1.11.1.x)

**Fig. 5:**
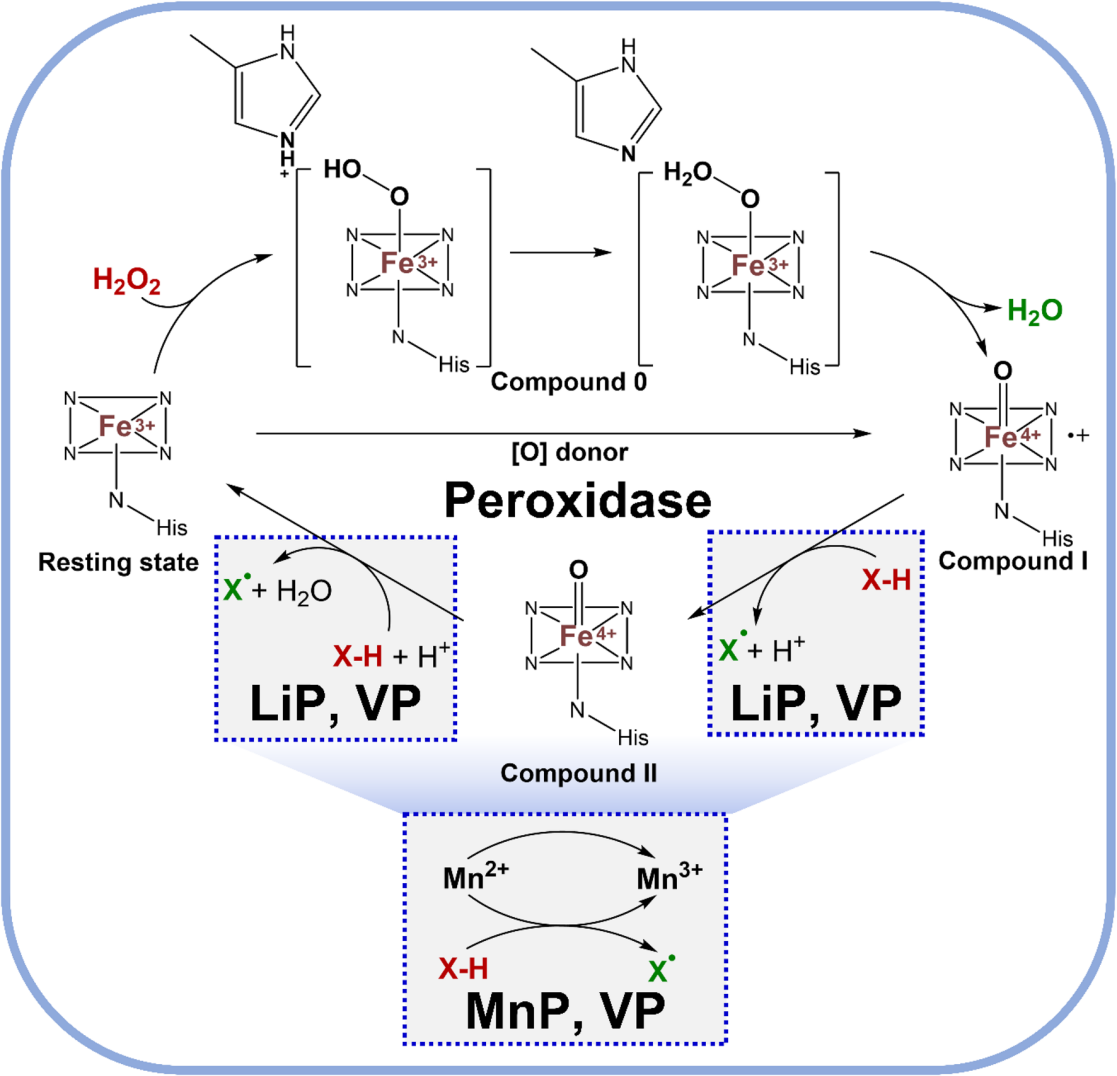
General mechanism of peroxidases (*LiP* lignin peroxidase, *MnP* manganese peroxidase, *VP* versatile peroxidase, *X*-*H* substrate). The free radical product X^•^ non-specifically reacts with other phenolic compounds

Lignin peroxidase (LiP), manganese peroxidase (MnP) and versatile peroxidase (VP) are three types of peroxidases that are involved in lignin degradation produce from various white-rot fungi and play a key role in fungal xenobiotic metabolism (Khan and Murphy 2023a). Although mechanisms of peroxidases have some variations, they share common features involving hydrogen peroxide (H_2_O_2_) as a co-substrate, resting state, Compound 0, Compound I, and Compound II (Kumar and Chandra 2020). A general overview of the mechanism for peroxidases is shown in Fig. 5 and is initiated with H_2_O_2_ attachment to the haem forming the Fe^3+^-hydroperoxo complex, Compound 0. Oxidation of haem generates Compound I (Fe^4+^=O^+•^), which is a highly reactive species, and water. Compound I abstracts a hydrogen atom from the substrate (X-H) and forms a substrate radical (X^•^) and Compound II (Fe^4+^=O); the substrate radical can then undergo further reactions, such as radical coupling (e.g., lignin breakdown). Compound II is a less reactive form of the enzyme, which can be regenerated to its resting state by accepting electrons from reducing agents or by direct reduction using H_2_O_2_.

MnP is primarily involved in the oxidation of Mn^2+^ ions using H_2_O_2_ to generate Mn^3+^ ions, which are important for lignin degradation and ligninolytic system activation. Mn^3+^ ions in the presence of organic peroxides, can oxidize a substrate (X-H) by abstracting hydrogen atoms, forming free radicals (X^•^), and initiating lignin degradation reactions. Like LiP, the reaction of MnP with H_2_O_2_ generates Compound I, which can further participate in substrate oxidation. Compound II is formed as a result of Compound I reduction, and it can be regenerated to the resting state by accepting electrons. The mechanism of versatile peroxidase involves a combination of LiP-like and MnP-like activities, enabling it to act on a wider range of substrates including oxidation of both phenolic and non-phenolic substances.

The value of peroxidases in biodegradation lies in the substrate range than can be oxidised by Compound I, and these substrates include phenols, PAHs, pesticides, dioxins, EDCs, PCBs, industrial dyes, and xenobiotics (Bansal and Kanwar 2013; Falade et al. 2017). However, there are several limitations that hinder the large-scale implementation of these enzymes such a sensitivity to changes in pH, temperature, presence of inhibitors, high redox potential, requirement for Mn^2+^ (for MnP and VP) and requirement for acidic pH levels and limited reuse. Nevertheless, there are strategies to address these limitations, for example, Son et al. (2021) showed that the thermostability of LiPH8 at acidic pH was improved in a triple mutant (S49C/A67C/H239E), which resulted in extra disulfide and ionic salt bridges leading to 10-fold increase in half-life at pH 2.5 and 25 °C.

Immobilisation is a strategy commonly used to improve operational stability of enzymes, longevity and ease of recycling (Gao et al. 2022) and recent examples include MnP from *A. flavus* that was immobilised on iron nanoparticles, and which displayed improved thermal stability, was active at a broader pH and temperature range, and showed improved decolorising activity compared with the non-immobilised enzyme (Kalsoom et al. 2022). Siddeeg et al. (2020) immobilised MnP from *Anthracophyllum discolor* on Fe_3_O_4_/chitosan nanocomposite and found that after five cycles of dye removal (methylene blue and reactive orange 16) activity was retained.

### Peroxygenases (EC 1.11.2.1)

**Fig. 6:**
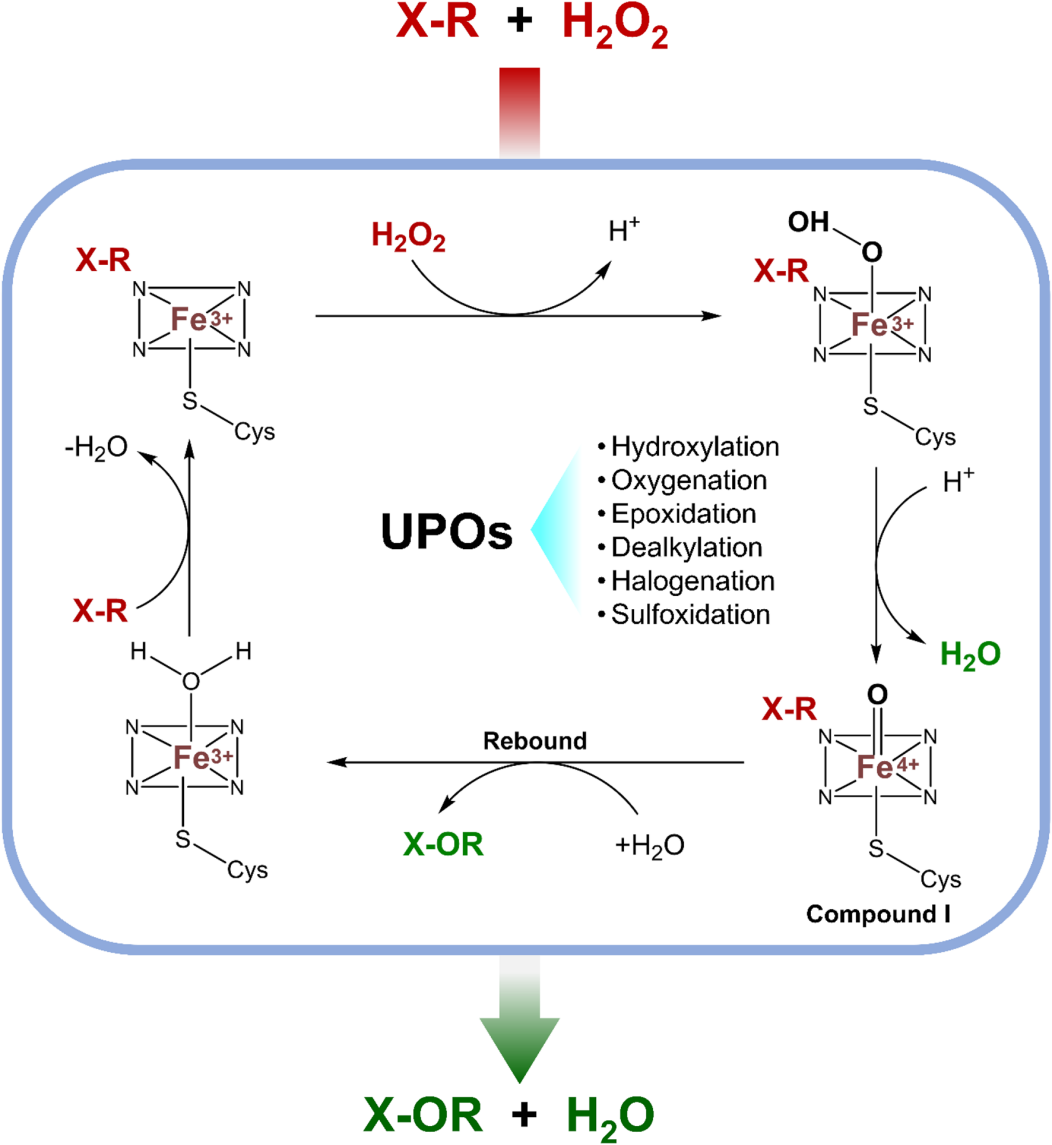
Mechanism of UPO

Fungal unspecific peroxygenases (UPOs) are versatile enzymes first identified in the fungus *Agrocybe aegerita* (Ullrich et al. 2004). Other UPOs were subsequently discovered in other mushrooms such as *Coprinellus* and *Marasmius* (Faiza et al. 2019). UPOs form a distinct subclass of peroxidases and belong to a separate superfamily of haem proteins and exhibit a range of catalytic activities (Fig. 6), often resembling the reactions carried out by CYPs but without an external electron donor (Rotilio et al. 2021). They function as a mono-peroxygenase by transferring an oxygen atom from hydrogen peroxide to various organic substrates, including aromatic, aliphatic and heterocyclic compounds (Hofrichter et al. 2015). H_2_O_2_ binds to the active site haem forming Compound 0 and then Compound I, as with peroxidases. Compound I reacts with the substrate generating a radical and hydroxylated Compound II and a rebound mechanism transfers oxygen to the substrate radical, returning the enzyme to its resting state (Hofrichter et al. 2022).

UPO efficiently catalyses a wide range of oxygenation reactions, making it a valuable tool for selectively oxidizing diverse compounds, including pollutants and recalcitrant molecules. Notably, UPO from *A. aegerita* can biotransform drugs such as propranolol, tolbutamide, acetanilide, naproxen and sildenafil to the human-equivalent metabolites (Poraj-Kobielska et al. 2011). Furthermore, when the UPOs from *A. aegerita* and *Marasmius rotula* were screened for their activity towards organic EPA priority pollutants they could transform 35 out of the 40 compounds tested (Karich et al. 2017). However, one major drawback for the application of UPOs is that H_2_O_2_, which is required as a substrate, also deactivates the enzymes. Thus, addition of H_2_O_2_ must be controlled, which is a challenge. Freakley et al. (2019) employed gold-palladium nanoparticles to generate H_2_O_2_ from H_2_ and O_2_ in situ, which was then used by the UPO PaDaI (evolved from *A. aegerita* UPO) to oxidise a range of substrates such as cyclohexane and ethylbenzene. Immobilisation can dramatically improve performance of UPO, for example, when the enzyme was immobilised in polyvinyl alcohol/polyethylene glycol beads resulted in a 60-fold improvement in total turnover number with diclofenac as the substrate (Poraj-Kobielska et al. 2015). Furthermore, when the beads were stored in an organic solvent (cyclohexane), not only was the enzyme activity preserved, but the relative activity improved.

### Laccase (EC 1.10.3.2)

**Fig. 7:**
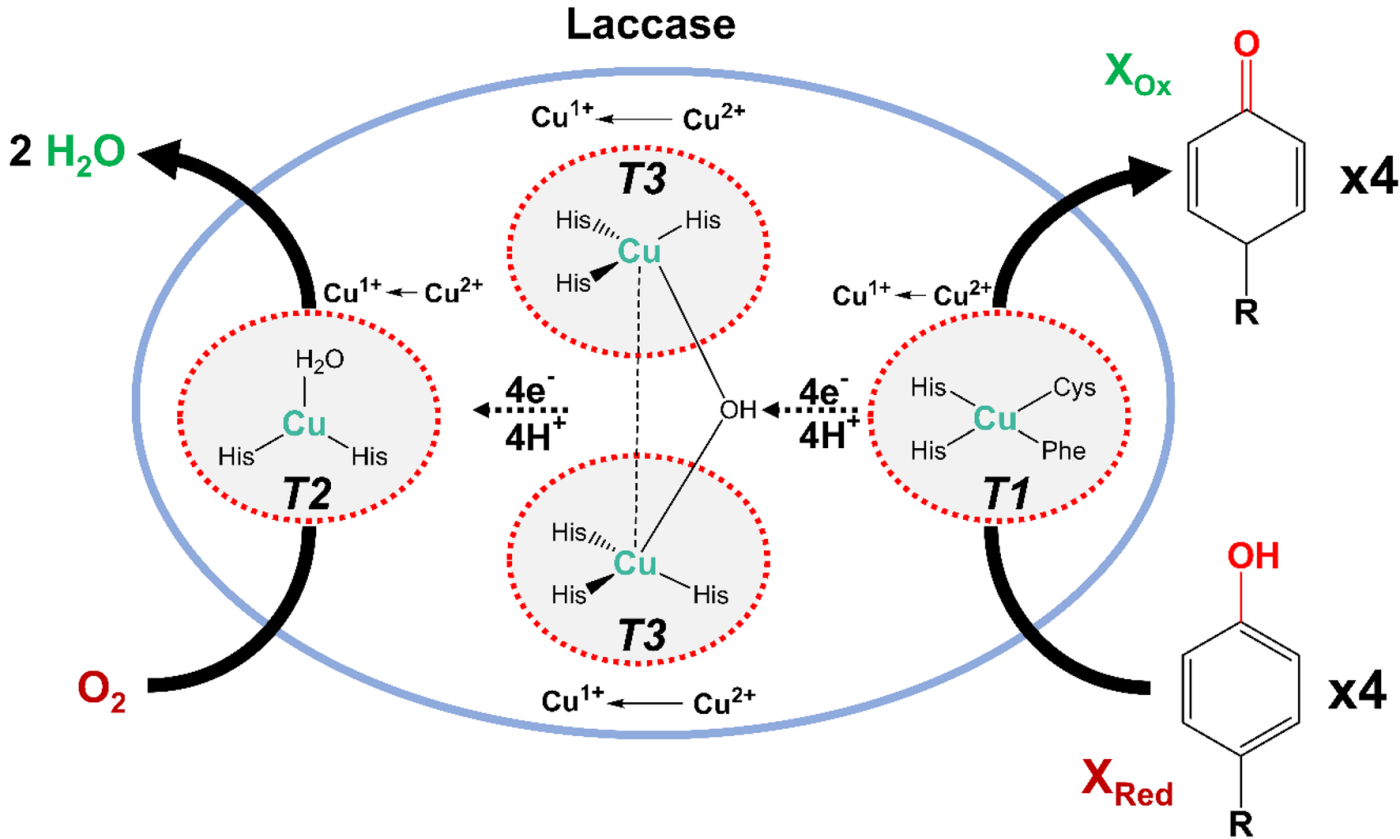
Mechanism of laccase. For each O_2_ molecule, four phenolic substrates are oxidised

Laccases are widely produced by various microorganisms, particularly white-rot fungi, to degrade lignin. They are extensively studied multi-copper oxidases with broad substrate specificities that oxidize various compounds, including phenols, polyphenols, aromatic amines and non-phenolic substrates via one-electron oxidations (Mayolo-Deloisa et al. 2020). This makes laccases valuable for several industrial applications including textile-dye bleaching and decolorization, detoxification of effluents, bioremediation and biofuel production. Laccases and peroxidases catalyze similar reactions but unlike peroxidases (LiP, VP and MnP), laccases have a lower oxidation potential with the advantage of using O_2_ as the electron acceptor. This eliminates the need for H_2_O_2_ addition, which although required by peroxidases, also inactivates them (Balcázar-López et al. 2016). The fungal laccase from *Trametes versicolor* contains four copper atoms in three centres in its active site: T1 (ligated by two His, one Cys, one Phe), T2 (ligated by two His), and the dicopper T3 (ligated by three His). In the process of catalysis (Fig. 7), the T1 copper initially accepts electrons from the substrate, then transferring them to the trinuclear copper cluster of T2 and T3. This trinuclear cluster plays a crucial role in the catalytic activity of laccases and is responsible for the reduction of O_2_ and the subsequent release of water with formation of a peroxo/peroxide transient intermediate (Rodríguez-Delgado et al. 2015; Tromp et al. 2010).

In spite of laccases’ industrial potential, there are certain constraints restricting application, including stability, reduced tolerance to high temperatures, alkaline pH, the need for expensive stimulators, low yield from native sources and challenges in the purification process (Agrawal et al. 2018). Heterologous expression offers the potential to achieve higher yields of laccase enzymes and enables the production of laccases with desired stability and catalytic properties specifically tailored for industrial applications. For example, Kurniati et al. (2022) reported that thermostability and pH stability of the laccase Lcc2 from *Pleurotus salmoneostramineus* improved when the gene was fused with one coding for a carbohydrate binding module and heterologously expressed in *S. cerevisiae*. Immobilisation of laccases from *T. versicolor* on chestnut biochar improved activity towards PAHs compared with the free enzymes, and the immobilised enzyme displayed higher stability upon storage and was active at a broader pH range and at higher temperatures (Zhao et al. 2023).

### Tyrosinase (EC 1.14.18.1)

Tyrosinase (or polyphenol oxidase) is a copper-containing enzyme present in microbes, plant and animals. Tyrosinase and laccase differ in their ability to oxidize substances, unlike laccase which has a broader range of substrate specificity, tyrosinase specifically oxidizes phenolic substrates, e.g., L-tyrosine (Muniraj et al. 2021). Like laccases, they are commercially produced from fungal sources, particularly through the cultivation of *Neurospora crassa* and *Agaricus bisporus*.

**Fig. 8:**
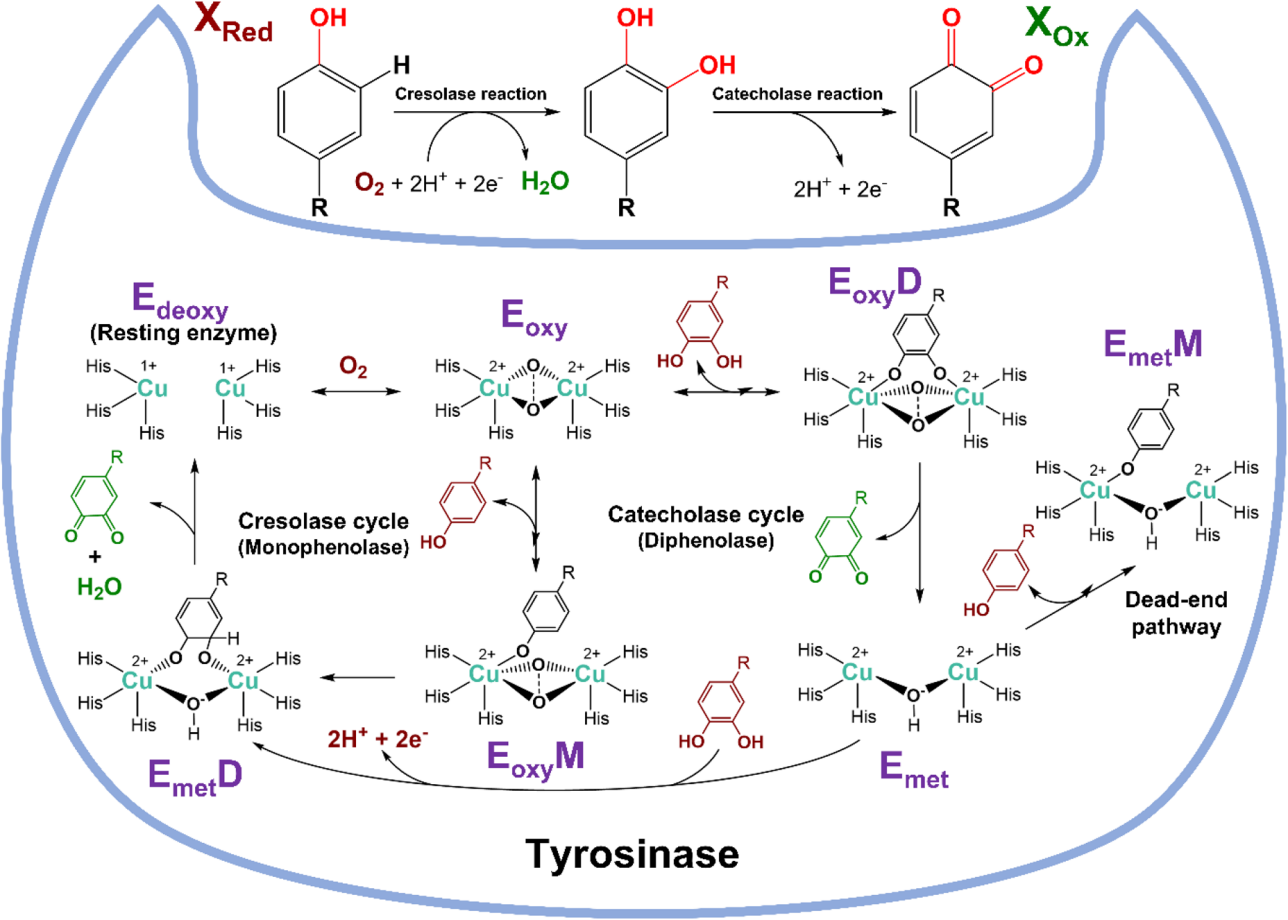
Catalytic mechanism of tyrosinase showing both cresolase and catecholase cycles. E_oxy_/E_met_/E_deoxy_ forms of oxidation state of tyrosinase. *E*_*oxy*_*M* monophenolase E_oxy_ complex, *E*_*oxy*_*D* diphenolase E_oxy_ complex, *E*_*met*_*M* monophenolase E_met_ complex, and *E*_*met*_*D* diphenolase E_met_ complex. A description of the mechanism is in the main text

The crystal structure of fungal tyrosinase has been solved and comprises a binuclear copper-active site in the deoxy-state (E_deoxy_), with each Cu^1+^ ion coordinated with three His residues (Ismaya et al. 2011). The tyrosinase catalytic mechanism involves the oxidation of phenolic substrates leading to the formation of o-diphenols (monophenolase or cresolase activity) and subsequent conversion to o-quinones (dihydroxyphenolase or catecholase activity) (Fig. 8). The reaction mechanism involves three main states, including E_oxy_ (oxy-tyrosinase; Cu^2+^–O_2_–Cu^2+^), E_met_ (met-tyrosinase; Cu^2+^–O–Cu^2+^), and E_deoxy_ (deoxy-tyrosinase; Cu^+^–Cu^+^). E_oxy_D, E_oxy_M, E_met_D and E_met_M are E_oxy_-Diphenol, E_oxy_-Monophenol, E_met_-Diphenol and E_met_-Monophenol complexes, respectively that form during the catalytic cycle of tyrosinase (Halaouli et al. 2006). E_oxy_ plays a crucial role in the initial steps of the tyrosinase mechanism which represents the state where molecular oxygen (O_2_) is bound to the copper ions in the active site leading Cu^+^ to Cu^2+^ oxidation and are bridged by an O_2_ molecule as Cu^2+^–O_2_–Cu^2+^. E_oxy_M is formed when the E_oxy_ state reacts with monophenolic substrates, resulting in the formation of o-diphenols and a water molecule. E_oxy_D is formed when the E_oxy_ state reacts with o-diphenols, leading to the formation of o-quinones and a water molecule. E_met_ is an intermediate state that is formed when one oxygen atom dissociates from the bound O_2_ in the E_oxy_ state and one of the copper ions is coordinated to a hydroxide ion (OH^−^) instead of an oxygen atom, resulting in a bridging hydroxyl group as Cu^2+^–O–Cu^2+^). E_met_D is formed when the E_met_ state reacts with diphenolic substrates, leading to the release of two H^+^ ions and two electrons whereas, E_met_M is formed when the E_met_ state reacts with monophenolic substrates, leading to the formation of o-diphenols and the regeneration of E_met_. E_deoxy_ represents the reduced state of tyrosinase which is formed when E_met_ reacts with o-diphenols, leading to the formation of o-quinones and the reduction of the copper ions from Cu^2+^ to Cu^+^ which involved in the final steps of the tyrosinase mechanism. The specific details of the mechanism can vary depending on the specific enzyme and the nature of the substrate being oxidized (Chang 2009).

Fungal tyrosinases have potential for bioremediation of wastewater contaminated with phenolic pollutants (Kameda et al. 2006; Sharma et al. 2021; Xu et al. 2011) and dyes (da Silva et al. 2013). However, often the role of tyrosinase in xenobiotic degradation is inferred from whole cell studies with the target compound and separate measurement of the enzyme activity using a substrate such as catechol. For example, Govindwar et al. (2014) reported the degradation of the azo dye Reactive Yellow 84 A by *Galactomyces geotrichum* and concluded that tyrosinase was involved by correlating its activity with dye degradation, but without demonstrating direct biotransformation.

### Outlook

#### Bioremediation

The examples of pollutants that are catabolised by fungi provided in this review and elsewhere indicate the potential that fungi have in the bioremediation of contaminated sites. Additionally, compared to other microorganisms, they have extensive mycelial networks which allows them to spread through solid matrices (Kadri et al. 2017; Maqbool et al. 2016). An example of an in situ bioremediation of polluted soil sing fungi was reported by Stella et al. (2017) who investigated the bioaugmentation of PCB-contaminated soil by *P. ostreatus* and *Irepx lacteus* and found up 50% removal of PCBs in dumpsite soils after 12 weeks. Furthermore, microbial community analysis revealed that *P. ostreatus* efficiently colonises the soil and appeared to encourage the growth of bacterial PCB-degraders. Many fungi have mutually beneficial relationships with plant roots, known as endo- and ecto-mycorrhizal associations. This association confers a survival advantage upon the fungi, enhancing their resistance to environmental stressors and resulting in improved inoculum performance when utilized in contaminated sites (Passatore et al. 2014). One recent example of the benefits of plant-fungal symbiosis was reported by Fu et al. (2022) who studied the impact of the relationship between endophytic fungus *Phomopsis liquidambaris* and rice on phenanthrene absorbed by the plant. The content of phenanthrene in the soil and rice (including leaves, roots, and grains) of the plant-endophyte interaction system was about 42% and 27% lower than that of the non-inoculated treatment.

Despite the apparent advantages of fungi in bioremediation application, there are barriers to implementation, not least the toxic effect of different pollutants, or their metabolites, on fungal activity. For example, the biodegradation of 6:2 FTOH by *C. elegans* is inhibited by 5:3 fluorotelomer carboxylic acid (FTCA), which is a fluorometabolite produced as a result of catabolism (Khan and Murphy 2023b), thus by itself the fungus is not a realistic means of remediating environments contaminated with this compound. A combination of approaches is one potential mechanism to ameliorate the effects of inhibition. For example, by combining sequential photocatalysis and fungal treatment (Khan et al. 2023) were able to improve the degradation of perfluorooctanoic acid (PFOA). It was suggested that the photocatalysis step reduced the initial concentration of the PFOA without producing the inhibitory 5:3 FTCA, allowing the fungus to remain active for longer, thus leading to an overall improved degradation of the substrate. A more complete understanding of the enzymatic steps involved in PFAS catabolism in fungi is required to enable optimal degradation, which should be the focus of future studies.

Similar synergistic approaches, in which fungi (or their enzymes) are combined with another chemical or biological methods, are likely to be a major part of pollution removal in the future. Fenton treatment of pollutants, in which the hydroxy radicals produced from Fe^2+^ and hydrogen peroxide react with organic compounds, is well established, but the high H_2_O_2_ consumption, acidic pH and production of high concentrations of iron-hydroxide are limitations. Some researchers have sought to combine the quinone recycling system of white rot fungi, which involves the lignin-degradaing enzymes laccase, Mn-peroxidase and lignin peroxidase, and results in in the production of hydrogen peroxide and hydroxyl radicals, and the reduction of Fe^3+^, with the Fenton reaction to promote pollution degradation (Chen et al. 2023).

### Biocatalysis

Fungal metabolism of xenobiotics can also be applied in a productive capacity. While the removal of pollutants via fungal catabolism is an important application on its own, some of the metabolites formed can be valuable. For example, Patil et al. (2021) reported that the main metabolite arising from chloropyrifos biotransformation in *Trametes hirsuta* MTCC-1171 is 2,4-bis (1,1 dimethylethyl) phenol, which is an important fuel additive. Lu et al. (2021) demonstrated that mushroom tyrosinase immobilised in a metal-organic framework (MOF) was a much-improved biocatalyst compared with the non-immobilised enzyme for production of the pharmacologically important catechols L-DOPA and hydroxytyrosol.

One other important application of xenobiotic metabolism is the production of mammalian drug metabolites that are required for toxicity testing and as standards. These compounds can be chemically synthesised or isolated from dosed animals, neither of which are ideal methods. However, as described in this review, fungal biotransformation of drugs can yield human-equivalent metabolites in good yields, and without the ethical or environmental concerns of the other methods. Furthermore, some researchers have explored the use of fungal biofilms to improve productivity by enabling easy re-use of the biocatalyst (Amadio et al. 2013; Bianchini et al. 2021; Quinn et al. 2015). The key enzymes responsible for drug metabolism in fungi are the CYPs. However, the inclusion of fungal CYPs in panels that are commercially employed to produce drug metabolites is hampered by lack of access to the coding sequences and knowledge of the function of the myriad CYPs encoded in some fungal genomes, for example, the CYPome of *P. chrysosporium* consists of almost 150 CYPs (Syed and Yadav 2012). Furthermore, heterologous expression of fungal CYPs can be difficult as they are membrane bound and require a suitable redox partner, which can be highly specific for the CYP and require co-expression with the host (Khan and Murphy 2022). Future implementation of fungal CYPs in drug metabolite production will require that these technical barriers are lowered.

An alternative to CYPs for drug metabolite production is fungal UPOs, which employ H_2_O_2_ as the oxidant, do not require a specific redox partner and can be tailored to produce a particular metabolite. For example, Gomez de Santos et al. (2018) engineered the *Agrocybe aegerita* UPO to produce 5’-hydroxypropanolol, using a structure-guided evolution approach. The evolved biocatalyst produced the desired metabolite with 99% regioselectivity.

In conclusion, fungal xenobiotic catabolism holds significant potential for applications in bioremediation and biocatalysis; however, the challenge is in translating the knowledge gained through basic research into marketable products. A focus on a deeper understanding the specific enzymes that are involved in fungal biotransformation of xenobiotics, factors that impact on their activity and improving the methods for their heterologous expression will facilitate their broader application.

## Data Availability

The datasets generated during and/or analysed during the current study are available from the corresponding author on reasonable request.
